# *Bacillus pumilus* probiotic feed supplementation mitigates *Lawsonia intracellularis* shedding and lesions

**DOI:** 10.1186/s13567-019-0696-1

**Published:** 2019-10-22

**Authors:** Tanja Opriessnig, Anbu K. Karuppannan, Dana Beckler, Tahar Ait-Ali, Ana Cubas-Atienzar, Patrick G. Halbur

**Affiliations:** 1 The Roslin Institute and The Royal (Dick) School of Veterinary Studies, University of Edinburgh, Easter Bush, Midlothian, EH25 9RG UK; 2 Department of Veterinary Diagnostic and Production Animal Medicine, College of Veterinary Medicine, Iowa State University, Ames, IA USA; 3 Gut Bugs Inc., Fergus Falls, MN USA

## Abstract

The causative agent of ileitis, *Lawsonia intracellularis,* is commonly associated with diarrhea and reduced weight gain in growing pigs. The effect of in-feed probiotics on *L. intracellularis* infection dynamics was evaluated. In brief, 70 2.5-week-old-pigs were randomly divided into six groups with 10–20 pigs each. All pigs were fed an age appropriate base ration for the duration of the study, which was supplemented with one of three *Bacillus* strains including *B. amyloliquefaciens* (T01), *B. licheniformis* (T02) and *B. pumilus* (T03). Another group was orally vaccinated with a commercial live *L. intracellularis* vaccine (VAC) at 3 weeks of age. At 7 weeks of age, T01-LAW, T02-LAW, T03-LAW, VAC-LAW and the POS-CONTROL groups were challenged with *L. intracellularis* while the NEG-CONTROL pigs were not challenged. All pigs were necropsied 16 days later. By the time of inoculation, all VAC-LAW pigs had seroconverted and at necropsy 10–65% of the pigs in all other challenged groups were also seropositive. The results indicate a successful *L. intracellularis* challenge with highest bacterial DNA levels in POS-CONTROL pigs, VAC-LAW pigs and T01-LAW pigs. There was a delay in onset of shedding in T02-LAW and T03-LAW groups, which was reflected in less severe macroscopic and microscopic lesions, reduced intralesional *L. intracellularis* antigen levels and a lower area under the curve for bacterial shedding. Under the study conditions, two of the probiotics tested suppressed *L. intracellularis* infection. The obtained findings show the potential of probiotics in achieving antibiotic-free control of *L. intracellularis*.

## Introduction

*Lawsonia intracellularis,* an obligate intracellular bacterium [1, 2], is the causative agent of ileitis in pigs [3] and infection of pig herds with *L. intracellularis* infection is commonly associated with economic losses [4]. Clinical signs may include diarrhea, reduced weight gain, decreased feed efficiency, and increased (highly variable) mortality rates [5–7]. *L. intracellularis* infections often are exacerbated by co-infecting pathogens [7, 8]. The disease course can be acute or chronic [7]. It is well recognized that pigs can also be infected subclinically [9, 10].

*Lawsonia intracellularis* is transmitted among pigs by the oral-fecal route. The organism has been shown to survive in the environment for up to 2 weeks in a temperature range of 5–15 °C [11]. It has been established that 10^4^–10^6^ organisms are sufficient to infect a pig [5, 10]. Infected pigs, including those subclinically-infected, actively shed the organism for 12 weeks or longer [11]. Transmission between herds may occur via contaminated equipment, insects or rodents [7, 12].

Control of *L. intracellularis* infection is mainly accomplished by treatment with antibiotics such as tiamulin, tylosin, chlortetracycline, lincomycin and olaquindox [6, 13], which are typically administered as standard doses approved by regulatory agencies in each country. These antibiotics are also used prophylactically and have been shown to reduce clinical signs, histological lesions and fecal shedding of the organism. An oral live vaccine and an inactivated parenteral vaccine are licensed for use in commercial pig production and are known to elicit humoral and cell mediated immunity [11, 14–17]. Vaccination does not completely prevent *L. intracellularis* shedding and is therefore occasionally used in combination with antibiotics [18]. In addition, an antibiotic-free feeding window is required for successful administration of live vaccine. However, with the increase in voluntary and mandatory antibiotic free pig husbandry practices, the control of *L. intracellularis* infection poses challenges for producers and veterinarians.

Probiotic bacteria are widely used in human nutrition. Major proposed probiotic mechanisms include competitive exclusion of pathogenic microorganisms, enhancement of the epithelial barrier, modulation of the immune system and others. Usage of probiotics is considered by many as an alternative to prophylactic antibiotic *in*-feed supplementation [19]. Several species of bacteria, such as *Bacillus* spp., are considered to have probiotic potential [20]. The objective of this study was to compare the effect of *Bacillus amyloliquefaciens*, *Bacillus licheniformis* and *Bacillus pumilus* on *L. intracellularis* infection. The results were directly compared to those obtained with a commercial live *L. intracellularis* vaccine.

## Materials and methods

### Animals and housing

**Figure 1:**
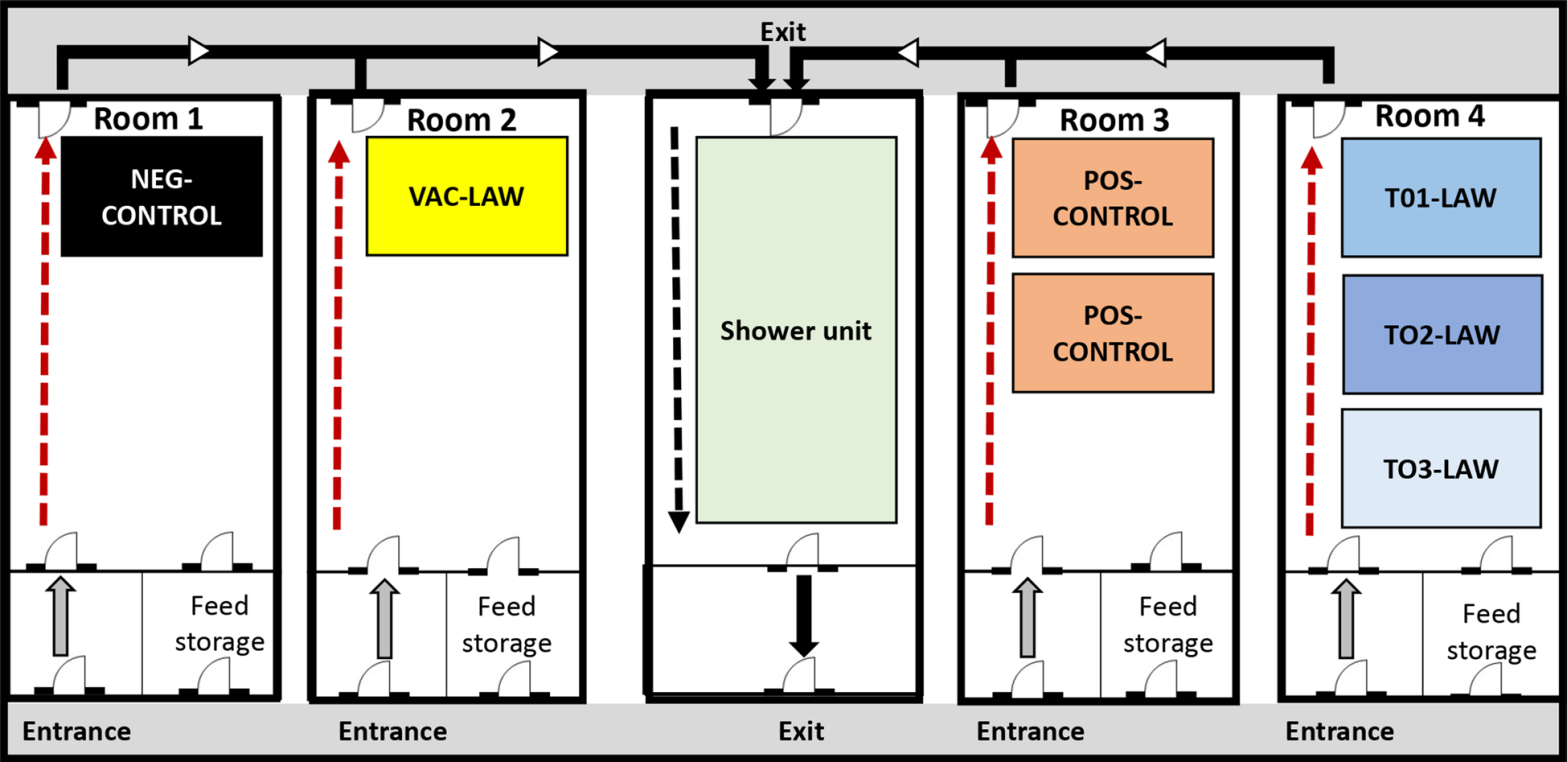
**Room and pen layout for the study.** Dashed or solid arrows indicate the flow direction of animal caretakers and research personnel in each treatment room to avoid room-to-room contamination.

Seventy 2.5-week-old age crossbred pigs were purchased from a *L. intracellularis*-free source herd where the dams were regularly tested and found to be negative for *L. intracellularis* antibodies using a commercial ELISA (SVANOVIR^®^ *L. intracellularis*/Ileitis-Ab, Svanova, Uppsala, Sweden). The pigs were transported to the Livestock Infectious Disease Isolation Facility at Iowa State University and randomly allocated into six experimental groups housed in four separate BSL-2 rooms with 1–3 pens (approximately 10 m^2^ each) of 10 pigs each equipped with a nipple waterer and a self-feeder (Figure 1). Specifically, the T01-LAW, T02-LAW and T03-LAW were housed in a single room with three separate pens approximately 2 m apart from each other (Figure 1).

### Experimental design and sample collection

**Figure 2:**
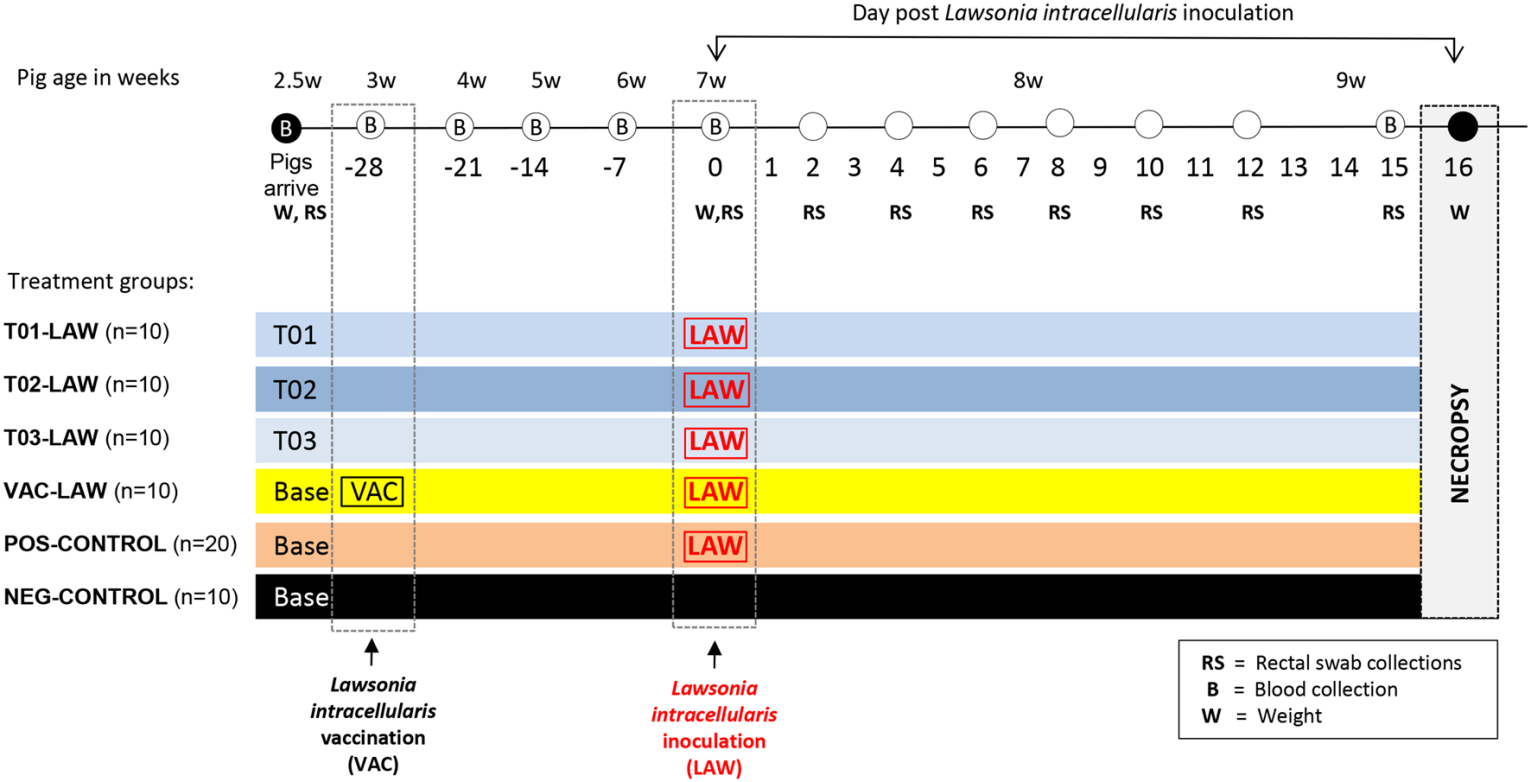
**Experimental design including time lines and major sampling events.** Base, base diet; T01, base diet with *Bacillus amyloliquefaciens*, TO2, base diet with *Bacillus licheniformis*, and T03, base diet with *Bacillus pumilus.*

At arrival, the pigs were randomly assigned to six treatment groups with 10–20 pigs each (Figure 2). At 3 weeks of age, the pigs were either vaccinated with a commercial oral vaccine against *L. intracellularis* (VAC-LAW), were supplied feed supplemented with one of the three probiotics (T01-LAW, *B. amyloliquefaciens*; T02-LAW, *B. licheniformis*; T03-LAW, *B. pumilus*), or remained non-treated (NEG-CONTROL, POS-CONTROL). At 7 weeks of age all groups except the NEG-CONTROL group were challenged with gut homogenate containing *L. intracellularis* by gastric gavage. Blood samples were collected from all pigs on a weekly basis. Rectal swabs (in 1 mL saline) were collected at arrival of the pigs at the research facility, prior to inoculation and at day post-inoculation (dpi) 2, 4, 6, 8, 10, 12 and 15.

### Feed

The feed used in this study was produced by a local feed mill (Heartland Co-Op, Prairie city, IA, USA) without addition of antibiotics or animal proteins. Two different regular base diets, HLN2 and HLN3, were fed to the pigs from 2.5 to 6 weeks (HLN2) and 6 to 9 weeks (HLN3; Table 1). A portion of the base diet was supplemented in the feed mill with 1 × 10^12^ colony forming units (CFU) per metric ton of *Bacillus amyloliquefaciens* (T01; 40.9 g/ton), *Bacillus licheniformis* (T02; 16.7 g/ton) or *Bacillus pumilus* (T03; 23.7 g/ton). Each different feed batch was color coded, labeled with Base, T01, T02 or T03, transported to the Iowa State University research facility and stored at 22 °C in a dark, dry storage room. The room temperature in the storage room was monitored daily. The pigs were fed *ad libidum* for the duration of the study with their allocated feed (Base, T01, T02 or T03) as outlined in Figure 2.

**Table 1:** **Base feed composition of the growth phase diets used**

Ingredient	HLN2 2.5–6 weeks of age	HLN3 6–9 weeks of age
Moisture %	10.5	11.3
Fat %	5.5	5.5
Crude fiber %	2.6	2.7
Calcium %	0.72	0.6
Phosphorus %	0.65	0.56
Salt %	0.7	0.55
Copper, added ppm	144	188
Iodine, added ppm	0.62	0.39
Iron, added ppm	119	132
Manganese, added ppm	38	32
Selenium, added ppm	0.26	0.24
Zinc, added ppm	2505	1572
Metabolicenergy, kcal/lb	1540	1557
Crude protein %	20.6	20.0
Lysine %	1.52	1.41
Digestible lysine %	1.37	1.27

### Vaccination

Pigs in the VAC-LAW group were vaccinated with a commercial *L. intracellularis* vaccine (Enterisol^®^ Ileitis, Boehringer Ingelheim, serial number 3040187B) at 3 weeks of age. This particular vaccine is a one dose product, contains a live bacterial culture, and is given orally. Prior to vaccination the vaccine was freshly reconstituted according to the manufacturer’s instructions and administered to each VAC-LAW pig by slowly dripping 2 mL of the vaccine with a syringe into the mouth.

### Inoculation

Inoculation was done when the pigs were 7 weeks old, 28 days post *L. intracellularis* vaccination, using an intestinal homogenate from an experimentally infected pig containing approximately 3 × 10^7^ *L. intracellularis* organisms per mL. The *L. intracellularis* inoculum was obtained from a commercial supplier (Gutbugs Inc, Fergus Falls, MN, USA). The *L. intracellularis* concentration in the inoculum was determined by quantitative PCR [21, 22]. The inoculum was tested previously and found to be negative for presence of different enteropathogens such as *Salmonella enterica*, enterotoxigenic *Escherichia coli*, oocysts or parasite eggs. In brief, the inoculum was thawed shortly prior to the inoculation and was transported/stored in several independent smaller containers on ice. Each pig was briefly restrained with a snare and 35 mL of the homogenate was administered by gastric gavage. The overall inoculation took approximately 45 min for all pigs. The order of inoculation was T03-LAW group first followed by T02-LAW, T01-LAW, POS-CONTROL pigs and finally the VAC-LAW pigs.

### Clinical assessment

The pigs were weighed at arrival (2.5 weeks of age), at inoculation (7 weeks of age, dpi 0) and at necropsy (9 weeks of age, dpi 16; Figure 2). In addition, fecal consistency was assessed on dpi 0, 2, 4, 6, 8, 10, 12 and 15. Specifically, the scores included 0 (normal, formed feces), 1 (semisolid feces), 2 (pasty feces), and 3 (watery/liquid feces).

### Necropsy

The pigs were euthanized by barbiturate overdose and presence and degree of macroscopic lesions were examined and scored by a veterinary pathologist (PGH) blinded to treatment group status. Specifically, ileum mucosal thinking was subjectively assessed using a score ranging from 0 (normal), 1 (mild), 2 (moderate), and 3 (severe). If present, the length of a given lesion was not assessed. However, to assess distribution by histopathology three sections of ileum (3–5 cm apart) and three sections of colon (3–5 cm apart) were collected from each pig. Samples from the ileum, colon and mesenteric lymph node were collected and as per standard research protocols frozen at −80 °C or fixed in 10% buffered formalin for histological analysis.

### Laboratory analyses

#### Serology

The *L. intracellularis*-specific antibody response was determined using the SVANOVIR^®^ *L. intracellularis*/Ileitis-Ab antibody test (Svanova, Uppsala, Sweden), a competitive ELISA, as per the manufacturer’s instructions. A 40% inhibition of the supplied standard positive serum was set as the criteria for a valid assay. A sample with more than 30% inhibition was considered positive, and a sample with less than 30% inhibition was considered negative.

#### DNA extraction

*Lawsonia intracellularis* DNA was extracted from the fecal swabs using the KingFisher Flex^®^ magnetic bead-based nucleic acid extraction platform and the MagMAX-96 nucleic acid isolation kit (ThermoFisher, Waltham, MA, USA).

#### Quantitative real-time PCR

A standard curve with a known amount of *L. intracellularis* was generated to establish the conditions for bacteria quantification initially. The copy numbers of *L. intracellularis* in the rectal swabs were estimated using a quantitative real-time PCR assay with the following primers and probe: forward primer, 5′-GCGCGCGTAGGTGGTTATAT-3′); reverse primer, 5′-GCCACCCTCTCCGATACTCA-3′; probe, 5′-FAM-CACCGCTTAACGGTGGAACAGCCTT-TAMRA-3′ [22]. The assay was performed in a ABI7500 Fast PCR machine with TaqMan Universal real-time PCR Master Mix (ThermoFisher, Waltham, MA, USA) using the following cycling conditions: initial incubation 50 °C for 2 min, incubation at 95 °C for 10 min, which was following by 40 cycling steps with 15 s at 95 °C and 1 min at 60 °C. A cycle threshold of 38 or greater was considered negative. Appropriate negative and positive controls were included in each extraction and real-time PCR run.

#### Histopathology

Microscopic lesions were assessed by a veterinary pathologist (TO) blinded to the treatment status. Intestinal sections including several sections of ileum and colon were scored for the presence of crypt epithelial hyperplasia characteristic of *L. intracellularis* infection (0, normal; 1, mild; 2, moderate; 3, marked with or without crypt herniation into the submucosa). While inflammation is not a prominent feature with *L. intracellularis* infection, the sections were also scored for presence of inflammation (0, normal; 1, mild cellular infiltrate; 2, moderate cellular infiltrate with or without submucosal infiltrate; 3, marked cellular infiltrate with or without submucosal infiltrate).

#### *L. intracellularis* immunohistochemistry

*Lawsonia intracellularis* antigen-specific IHC was performed on the intestinal sections as described [23]. The presence and amount of *L. intracellularis* antigen was scored and ranged from 0 (no signal), 1 (few focal crypts showing low numbers of enterocyte staining), 2 (few multifocal crypts showing low-moderate amounts of enterocytes with staining), to 3 (most crypts in most sections showing marked apical enterocyte labelling). To simplify the analysis, a combined ileum lesion score was introduced for which the scores for crypt enterocyte hyperplasia, inflammation and amount of *L. intracellularis* antigen were combined for a maximum score of 9. Using this combined ileum lesion score, the lesions were classified as absent (score of 0), mild (scores of 1, 2 or 3), moderate (scores of 4, 5 and 6) and severe (scores of 7, 8 and 9).

#### Periodic acid Schiff staining

For the Periodic acid Schiff (PAS) staining, fixed paraffin-embedded ileum sections from pigs were dewaxed and rehydrated according to standard protocols. Following this, sections were immersed in 1% periodic acid for 5 min and in Schiff’s reagent for 15 min as described [24]. Slides were then immersed in warm water to intensify the red staining of the goblet cells. The slides were counterstained with Haemalum Mayer (Sigma-Aldrich Company Ltd, Dorset, UK) for 5 min and dipped for 2 min with Scots tap water. The tissue sections were washed in running tap water and mounted with mounting medium (Fisher Scientific, Loughborough, UK).

#### Intestinal alkaline phosphatase staining

Intestinal alkaline phosphatase (IAP) was stained with Vector^®^ Red Alkaline Phosphatase Substrate (Vector Laboratories, Cambridge, UK) according to manufacturer instructions. Slides were counterstained with Haemalum Mayer and mounted as described for the PAS staining.

#### Quantitative image analysis

For quantitative analysis of PAS and IAP stained slides were scanned using NanoZoomer XR digital slide scanner (Hamamatsu Photonics, Hamamatsu, Japan). For PAS staining, images were captured using brightfield, and for IAP a combination of brightfield and Texas red filter was used. Digital images were acquired at 40× magnification and analyzed using open-access image analysis software ImageJ version 1.49 (National Institutes of Health, Bethesda, MD, USA). Staining was analyzed by quantifying the intensity of the entire sections and a total of three sections per staining were quantified. Briefly, the spectrally opposite color channel was selected, and the intensity threshold was adjusted to highlight goblet cells for PAS staining or the villi for IAP staining [16].

### Statistical analysis

The statistical analysis was performed using JMP software (JMP version Pro 13.1.0, SAS Institute Inc., Cary, NC, USA). Summary statistics were calculated to assess the overall quality of the data. Analysis of variance (ANOVA) was used for cross-sectional assessment of the average daily weight gain (ADG) and level of fecal shedding of *L. intracellularis*. Real-time PCR results (copies per fecal swab) were log_10_ transformed, demonstrated a normal distribution, and were then analyzed. The significance level was *P *< 0.05 followed by pairwise testing using the Tukey–Kramer adjustments to identify the groups that were different. Non-repeated measures of necropsy and histopathology data were assessed using non-parametric Kruskal–Wallis ANOVA. If a non-parametric ANOVA test was significant (*P* < 0.05), then Wilcoxon tests were used to assess the differences of pairs of groups. Differences in incidence were evaluated by using Fisher’s exact test. The area under the curve (AUC) of bacterial shedding of each animal and the total AUC for each group was calculated using the log transformed values of the bacterial loads over time. One-way ANOVA and a Bonferroni post hoc test were used to compare groups.

To determine a cutoff for *L. intracellularis* values of infected (T01-LAW, T02-LAW, T03-LAW, VAC-LAW and POS-CONTROL pigs) and non-infected pigs (NEG-CONTROL) based on PAS and IAP staining intensity in ileum sections, receiver operating characteristic (ROC) curve analysis SPSS statistics (Version 23.0. IBM Corp. Armonk, NY, USA) was used. The cutoff was calculated using the Youden Index. Correlations of PAS or IAP values with overall ileum lesion severity or *L. intracellularis* DNA shedding length in fecal swabs were also calculated and compared as described [25]. Specifically, DNA shedding length was done by counting consecutive *L. intracellularis* PCR positive rectal swabs over time in a given pig.

## Results

### *L. intracellularis*-specific antibody responses

At arrival in the research facility, all pigs were negative for *L. intracellularis* antibodies (data not shown). At the day of inoculation (28 days post-vaccination) all VAC-LAW pigs had seroconverted with a group mean percentage of inhibition value ± SEM of 53.1 ± 1.9 which was significantly (*P* < 0.05) higher compared to all other groups indicating successful vaccination of this group. The number of pigs that seroconverted within the 15 days between *L. intracellularis* inoculation and blood collection prior to necropsy was 4/10 in the T01-LAW group (group mean/SEM; 30.1 ± 6.9), 1/10 in the T02-LAW group (14.7 ± 2.4), 1/10 in the T03-LAW group (12.8 ± 4.2), 10/10 in the VAC-LAW group (44.5 ± 1.8), 0/10 in the NEG-CONTROL group (7.2 ± 1.6) and 13/20 in the POS-CONTROL group (33.9 ± 4.4).

### Clinical signs and average daily gain

There were no clinical signs in any of the pigs present prior to *L. intracellularis* inoculation. Pigs in the NEG-CONTROL group remained free of clinical signs throughout the study period. Individual pigs in all *L. intracellularis* groups had semisolid or pasty feces on single days throughout the study. Watery or bloody diarrhea (score 3) was present in 4/20 POS-CONTROL pigs and 1/10 VAC-LAW pigs by dpi 15. The average daily gain (ADG) before and after challenge is summarized in Table 2. After *L. intracellularis* infection, all infected groups had significantly less ADG compared to the NEG-CONTROL group without significant differences among *L. intracellularis* infected groups.

**Table 2:** **Average daily weight gain (ADG) in grams ± SEM before or after** ***L. intracellularis*** **infection**

Group	Before inoculation 2.5–7 weeks of age	After inoculation 7–9 weeks of age
T01-LAW	435.2 ± 21.3^A,a^	712.7 ± 32.2^A^
T02-LAW	408.0 ± 30.0^A^	665.3 ± 32.9^A^
T03-LAW	473.5 ± 12.3^A^	646.3 ± 21.1^A^
VAC-LAW	413.2 ± 18.4^A^	623.3 ± 34.0^A^
NEG-CONTROL	424.3 ± 19.8^A^	877.8 ± 32.8^B^
POS-CONTROL	451.5 ± 9.8^A^	627.2 ± 27.2^A^

^a^Different superscripts (A,B) indicate a significant different ADG (*P* < 0.05) among groups during a time period.

### *L. intracellular* shedding

**Figure 3:**
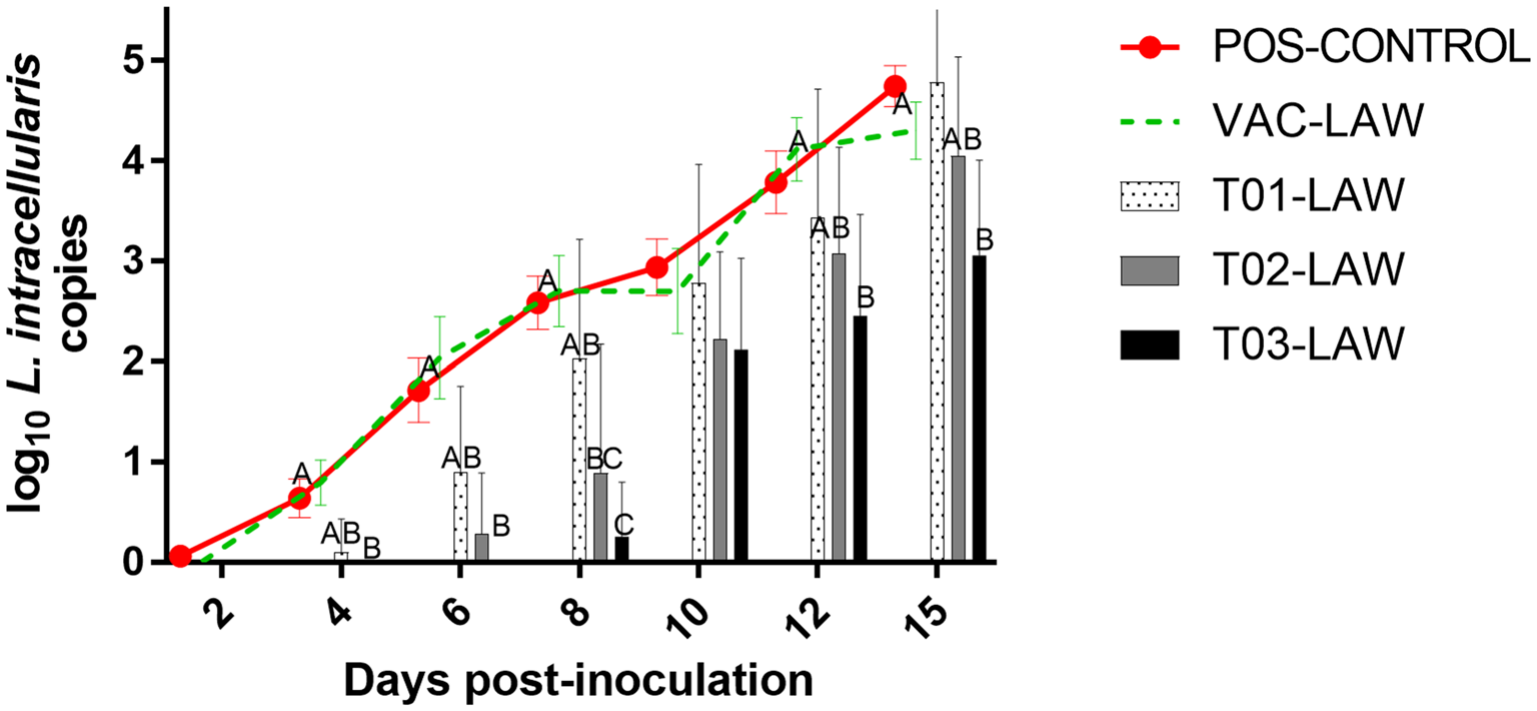
**Group mean log**_**10**_ ***L. intracellularis*** **DNA in rectal swabs over time.** Different superscripts (A,B,C) at a day post-challenge indicate a significant (*P *< 0.05) difference among groups.

**Figure 4:**
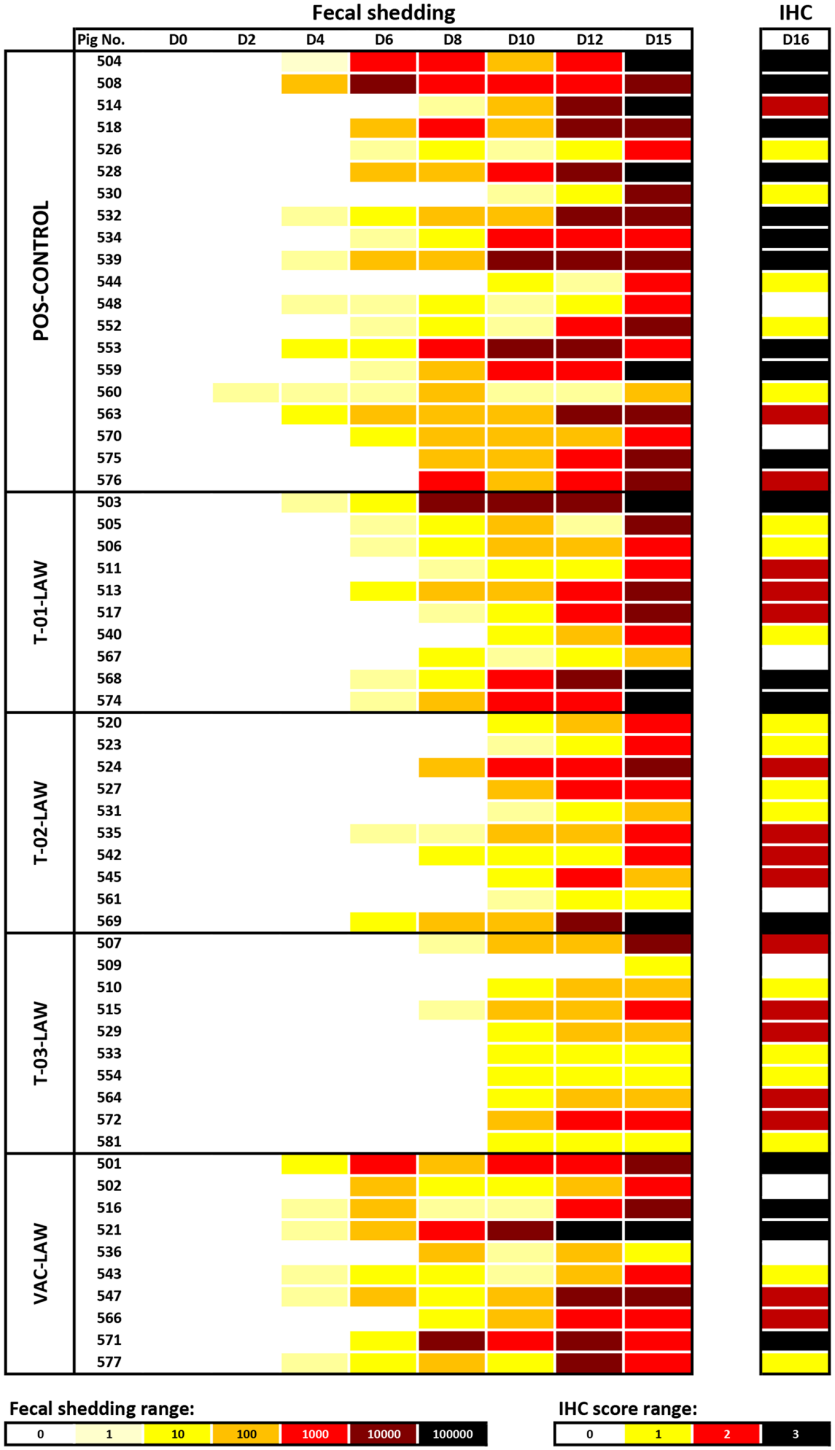
**Heat map outlining** ***L. intracelluaris*** **fecal shedding and tissue levels for each pig.** Fecal shedding was determined by *L. intracelluaris* PCR on rectal swabs and tissue levels were assessed at necropsy (D16) by immunohistochemistry on formalin-fixed ileum sections.

*Lawsonia intracellularis* DNA was never detected in rectal swabs of any of the NEG-CONTROL pigs for the duration of the study. *L. intracellularis* DNA was also not detected in any of the other groups at arrival at the research facility or on the day of inoculation. *L. intracellularis* shedding was first detected in a POS-CONTROL pig by dpi 2. The prevalence rates of *L. intracellularis* shedding in fecal swabs are summarized in Figure 3. There was a delay in shedding in the T02-LAW and T03-LAW groups compared to the POS-CONTROL and VAC-LAW groups with PCR positive pigs first detected at dpi 6 (2/10; T02-LAW) or dpi 8 (2/10; T03-LAW) (Figure 4). The prevalence of PCR positive pigs was significantly lower in T02-LAW and T03-LAW pigs compared to POS-CONTROL and VAC-LAW pigs on dpi 6 and 8. A summary of the group mean log genomic numbers of *L. intracellularis* for each dpi and the shedding length in weeks is presented in Table 3. For the duration of the trial (except dpi 10), pigs in the T03-LAW group shed significantly lower numbers of *L. intracellularis* compared to VAC-LAW and POS-CONTROL pigs. The AUC was highest and not different for T01-LAW, VAC-LAW, and POS-CONTROL while it was significantly (*P* < 0.05) reduced for T02-LAW and T03-LAW (Table 3).

**Table 3:** ***L. intracellularis*** **shedding characteristics**

Group	2	4	6	8	10	12	15	AUC	Shedding length
T01-LAW	0 (0)	0.1 ± 0.1^A,B,a^	0.9 ± 0.3^A,B^	2.0 ± 0.4^A,B^	2.8 ± 0.4^A^	3.4 ± 0.4^A,B^	4.8 ± 0.3^A^	11.6^A,B^	3.6 ± 0.3^A,B^
T02-LAW	0 (0)	0 (0)^A^	0.3 ± 0.2^A^	0.9 ± 0.4^A,C^	2.2 ± 0.3^A^	3.1 ± 0.3^A,B^	4.0 ± 0.3^A,B^	8.5^B,C^	2.6 ± 0.3^B,C^
T03-LAW	0 (0)	0 (0)^A^	0 (0)^A^	0.3 ± 0.2^C^	2.1 ± 0.3^A^	2.5 ± 0.3^A^	3.1 ± 0.3^B^	6.4^C^	2.0 ± 0.3^C^
VAC-LAW	0 (0)	0.8 ± 0.2^B^	2.0 ± 0.4^B^	2.7 ± 0.4^B^	2.7 ± 0.4^A^	4.1 ± 0.3^B^	4.3 ± 0.3^A^	14.5^A^	4.4 ± 0.3^A^
NEG-CONTROL	0 (0)	0^A^	0^A^	0^C^	0^B^	0^C^	0^C^	0^D^	0^D^
POS-CONTROL	5 (0.1 ± 0.1)	0.6 ± 0.2^B^	1.7 ± 0.3^B^	2.6 ± 0.3^B^	2.9 ± 0.3^A^	3.8 ± 0.3^B^	3.8 ± 0.2^A^	14.1^A^	4.1 ± 0.2^A^

Log group mean *L. intracellularis* genomic copies ± SEM in fecal swabs at different days post-infection (dpi), area under the curve (AUC) and shedding length in weeks.

^a^Different superscripts (A,B,C,D) indicate a significant different weight gain (*P* < 0.05) among groups during a time period.

### Macroscopic lesions

Macroscopic lesions were not observed in the NEG-CONTROL group while lesions consistent with *L. intracellularis* infection including hemorrhagic proliferative enteritis with inflammation, petechial hemorrhages, ulcers and thickening of the distal small intestine mucosa were observed in the other groups. Specifically, the mean group macroscopic ileum thickening based on subjective individual scores was 0.8 ± 0.3 for T01-LAW, 1.0 ± 0.1 for T02-LAW, 0.9 ± 0.1 for T03-LAW, 1.3 ± 0.3 for VAC-LAW and 1.9 ± 0.2 for POS-CONTROL pigs. The thickening was significantly reduced (*P* < 0.05) in the T01-LAW, T02-LAW and T03-LAW group compared to the POS-CONTROL pigs. Often the intestinal content was fluid and bloody with necrotic debris adhered to the mucosa. Liquid intestinal content (score 3) was present in 1/10 T01-LAW pigs, 0/10 T02-LAW pigs, 0/10 T03-LAW pigs, 1/10 VAC-LAW pigs and 8/20 POS-CONTROL pigs. In addition, mesenteric lymph nodes were often macroscopically enlarged.

### Microscopic lesions

Histological analysis of the intestinal tissue revealed lesions typical of *L. intracellularis* including crypt epithelial hyperplasia, increased numbers of mitotic figures throughout the crypt, depletion of goblet cells and presence of *L. intracellularis* antigen in enterocytes. The mean microscopic lesions are summarized in Table 4. Severe *L. intracellularis* associated lesions were identified in 30% of the T01-LAW pigs, 10% of the T02-LAW pigs, 40% of the VAC-LAW pigs and 55% of the POS-CONTROL pigs while severe lesions were not present in NEG-CONTROL or T03-LAW pigs. *L. intracellularis* antigen was identified in 9/10 T01-LAW pigs (0.7 ± 0.3), 9/10 T02-LAW pigs (0.3 ± 0.2), 9/10 T03-LAW pigs (0.2 ± 0.1), 8/10 VAC-LAW pigs (0.7 ± 0.2), 0/10 NEG-CONTROL pigs, and 18/20 POS-CONTROL pigs (1.2 ± 0.2). The *L. intracellularis* antigen levels were significantly (*P* < 0.05) higher in T01-LAW, VAC-LAW and POS-CONTROL pigs compared to NEG-CONTROL pigs, whereas there were significantly (*P *< 0.05) lower antigen amounts in T02-LAW and T03-LAW compared to POS-CONTROL pigs (Figure 4).

**Table 4:** **Group mean microscopic** ***L. intracellularis*** **lesion score**

Group	Mean lesion score (range: 0–9)	Lesion severity distribution			
		Normal	Mild	Moderate	Severe
T01-LAW	4.3 ± 0.9^A^	1	3	3	3
T02-LAW	3.3 ± 0.7^A^	1	8	0	1
T03-LAW	3.3 ± 0.5^A^	1	6	3	0
VAC-LAW	4.9 ± 1.1^A^	1	4	1	4
NEG-CONTROL	0.0 ± 0.0^B^	10	0	0	0
POS-CONTROL	5.6 ± 0.8^A^	2	5	2	11

The score is based on a combination of intestinal hyperplasia, inflammation and *L. intracellularis* antigen and distribution of the pigs in each group into normal (score 0), mild (score 1, 2 or 3), moderate (score 4, 5 or 6) or severe (score 7, 8 or 9).

^a^Different superscripts (A,B) indicate a significant different weight gain (*P* < 0.05) among groups during a time period.

### PAS and IAP staining and correlation with disease

PAS staining reveals the presence of mucopolysaccharides, glycoproteins, and/or glycogen and is traditionally used for the assessment of goblet cells, which are depleted during *L. intracellularis* infection [24]. The group mean PAS staining intensity was 2.2 ± 0.1 for NEG-CONTROLs, 1.3 ± 0.1 for T01-LAW, 1.4 ± 0.1 for T02-LAW, 1.5 ± 0.1 for T03-LAW, 1.4 ± 0.1 for VAC-LAW and 1.3 ± 0.1 for POS-CONTROL pigs. IAP, a brush-border enzyme, is an indicator of gut mucosal integrity and marker for crypt-villus differentiation [26]. The group mean IAP staining intensity was 5.5 ± 0.5 for NEG-CONTROL pigs, 1.2 ± 0.3 for T01-LAW, 1.7 ± 0.6 for T02-LAW, 2.6 ± 0.7 for T03-LAW, 2.3 ± 0.6 for VAC-LAW and 1.7 ± 0.5 for POS-CONTROL pigs. All groups infected with *L. intracellularis* had a significantly (*P* < 0.001) lower mean staining intensity compared to NEG-CONTROL pigs for both PAS and IAP.

PAS stain had an AUC of 0.9 ± 0.0. At a cutoff value of 1.6 the sensitivity was 100% and the specificity was 75%. Using this cutoff all NEG-CONTROL pigs were considered healthy while only 25% (15/60) of the *L. intracellularis* infected pigs were considered healthy. For the IAP stain, the AUC was 0.9 ± 0.0. At a cutoff value of 3.6 the sensitivity was 100% and the specificity was 83% and all NEG-CONTROL pigs were considered healthy, while 16.7% (10/60) of the *L. intracellularis* infected pigs were considered healthy. The correlation of PAS stain intensity with an ileum lesion severity score (a combined histopathology and immunohistochemistry score) was moderate (−0.536) and it was high (−0.752) for *L. intracellularis* shedding length. The correlation of IAP stain intensity with the ileum severity score was low (−0.467) and it was moderate with *L. intracellularis* shedding length (−0.672).

## Discussion

In this study, T02-LAW and T03-LAW groups showed a delayed *L. intracellularis* shedding compared to the POS-CONTROL group. The T02-LAW group was significantly different from the POS-CONTROL and the VAC-LAW group on dpi 6 and dpi 8. The T03-LAW group had significantly lower fecal shedding of *L. intracellularis* throughout the study period (except on dpi 10). In addition, microscopic lesions consistent with severe *L. intracellularis* infection (scores of 7, 8 and 9) were not seen in the T03-LAW pigs. Interestingly, pigs in groups T02-LAW and T03-LAW had a delayed or limited seroconversion against *L. intracellularis*, with only 10% of the pigs in the T03-LAW group seroconverting by dpi 15. This could reflect the delay in the colonization of the intestinal tissue by *L. intracellularis* in the T02-LAW and T03-LAW groups and thereby delay in the onset of humoral immune response against the pathogen. This is further corroborated by the observation that no fecal shedding could be observed in the T03-LAW group on dpi 6. Furthermore, on dpi 8, only 2/10 of the T03-LAW pigs were shedding compared to 18/20 POS-CONTROL pigs, indicating the degree of mitigation. Probiotic organisms *B. amyloliquefaciens* and *B. licheniformis*, fed to the groups T01-LAW and T02-LAW, respectively, were also effective in mitigating the shedding of the *L. intracellularis* albeit to a lesser degree than the *B. pumilus.* While *B. amyloliquefaciens* and *B. licheniformis* may also offer a certain level of protection against enteric pathogens in pigs and other livestock species [27], their antibacterial activity needs to be better characterized.

Numerous modes by which the *Bacillus* spp. mediate their probiotic action have been described [28]. Secretion of competence- and sporulation-stimulating factor by the *Bacillus* spp. has been shown to induce resistance to oxidative damage in host intestinal cells and improve their barrier function [29]. *Bacillus* spp. are also known to produce cyclic lipo-peptides which have a variety of activities including interactions with biofilms, and anti-fungal, anti-inflammatory, anti-tumor, anti-virus properties [30]. Surfactins, Iturins, Fengycins, Pumilacidins are families of such lipopeptides produced by many *Bacillus* spp. The lipopeptides are known to self-assemble and form micelles which have surfactant activity on biological membranes [31]. *Bacillus pumilus* is known to lyse cells of *Vibrio* spp. [32]. Polyketides are another family of compounds produced by the *Bacillus* spp. which have potent antibacterial activity, besides other biological activity [33]. The compounds produced by *Bacillus* spp. may have a direct effect on the *L. intracellularis* and may also prevent the colonization of enterocytes by *L. intracellularis* by inhibition of the pathogens host cell adhesion or entry.

Probiotics may act synergistically when used in combination, as their mode of action may be complementary to one another. Further, in spite of a challenge dose of 3 × 10^7^ *L. intracellularis* organisms and the intragastric inoculation route used in this study, the T03-LAW group had significantly lower fecal shedding of *L. intracellularis* at dpi 4, 6, 8, 12 and 15 compared to POS-CONTROL and VAC-LAW groups, and fewer and less severe, but not statistically different, gross and histopathological lesions than the VAC-LAW group. The *L. intracellularis* specific antibodies, especially IgA, induced by vaccines are thought to be important in mediating protection against infection of enterocytes by *L. intracellularis* and efforts to bolster the IgA in the ileal mucosa by feed supplementation with short-chain fructo-oligosaccharide have shown improved protection against *L. intracellularis* [15, 34]. In this study, total Ig antibody levels in serum without differentiation between IgM, IgG or IgA were measured. It may be interesting to determine IgA levels on the obtained samples to investigate if a lack of IgA could have been responsible for the results observed in the VAC-LAW pigs under the conditions of this study. The observations here suggest that *B. pumilus* may be able to prevent or limit the infection of enterocytes by *L. intracellularis* due to a yet unknown mechanism. This warrants further investigation especially under field conditions with the natural dynamics of infection, i.e., a lower but constant dose of infectious exposure at play. Identifying the mechanism by which *B. pumilus* inhibits *L. intracellularis*, a major primary pathogen in pig production around the world and also a risk factor for other enteric pathogens such as *Salmonella enterica* serovar Typhimurium [35, 36], would be a novel effort and support the cause of reducing antibiotics usage in food animal production.

The results of this study indicate that *B. pumilus* efficiently prevented early colonization of *L. intracellularis* in the pig enteric system as evidenced by reduced fecal shedding with a significantly lower AUC, even in the face of a high-dose experimental inoculation. The two other probiotics, *B. amyloliquefaciens and B. licheniformis,* displayed any anti-*L. intracellularis* effect to a lesser extent. This study warrants further detailed exploration of the potential of the *B. pumilus* to prevent or limit the impact of *L. intracellularis* infection in growing pigs.

## References

[1] Gebhart CJ, Barns SM, McOrist S, Lin GF, Lawson GH (1993). Ileal symbiont intracellularis, an obligate intracellular bacterium of porcine intestines showing a relationship to *Desulfovibrio* species. Int J Syst Bacteriol.

[2] Lawson GH, McOrist S, Jasni S, Mackie RA (1993). Intracellular bacteria of porcine proliferative enteropathy: cultivation and maintenance in vitro. J Clin Microbiol.

[3] McOrist S, Jasni S, Mackie RA, MacIntyre N, Neef N, Lawson GH (1993). Reproduction of porcine proliferative enteropathy with pure cultures of ileal symbiont intracellularis. Infect Immun.

[4] McOrist S (2005). Defining the full costs of endemic porcine proliferative enteropathy. Vet J.

[5] Vannucci FA, Gebhart CJ (2014). Recent advances in understanding the pathogenesis of *Lawsonia intracellularis* infections. Vet Pathol.

[6] Jacobson M, Fellstrom C, Jensen-Waern M (2010). Porcine proliferative enteropathy: an important disease with questions remaining to be solved. Vet J.

[7] McOrist S, Gebhart CJ, Zimmerman JJ, Karriker LA, Ramirez A, Schwartz KJ, Stevenson GW (2012). Proliferative enteropathy. 10^th^ diseases of swine.

[8] Komine M, Cunha TO, Mullaney TP, Smedley RC, Langohr IM (2016). Pathology in Practice. Proliferative and necrotizing enterocolitis in a pig resulting from coinfection with *L. intracellularis* and S. enterica. J Am Vet Med Assoc.

[9] Brandt D, Kaim U, Baumgartner W, Wendt M (2010). Evaluation of *Lawsonia intracellularis* infection in a group of pigs in a subclinically affected herd from weaning to slaughter. Vet Microbiol.

[10] Paradis MA, Gebhart CF, Toole D, Vessie G, Winkelman NL, Bauer SA, Wilson JB, McClure CA (2012). Subclinical ileitis: diagnostic and performance parameters in a multi-dose mucosal challenge model. J Swine Health Prod.

[11] Guedes RM, Gebhart CJ (2003). Onset and duration of fecal shedding, cell-mediated and humoral immune responses in pigs after challenge with a pathogenic isolate or attenuated vaccine strain of *Lawsonia intracellularis*. Vet Microbiol.

[12] Gabardo MP, Sato JPH, Daniel AGS, Andrade MR, Pereira CER, Rezende TP, Otoni LVA, Rezende LA, Guedes RMC (2017). Evaluation of the involvement of mice (*Mus musculus*) in the epidemiology of porcine proliferative enteropathy. Vet Microbiol.

[13] Jensen VF, Jorsal SL, Toft N (2017). A cross-sectional study of oral antibacterial treatment patterns in relation to specific diarrhoeal pathogens in weaner pigs. Vet Microbiol.

[14] Kroll JJ, Roof MB, McOrist S (2004). Evaluation of protective immunity in pigs following oral administration of an avirulent live vaccine of *Lawsonia intracellularis*. Am J Vet Res.

[15] Guedes RM, Gebhart CJ (2010). Evidence of cell-mediated immune response and specific local mucosal immunoglobulin (Ig) A production against *Lawsonia intracellularis* in experimentally infected swine. Can J Vet Res.

[16] Roerink F, Morgan CL, Knetter SM, Passat MH, Archibald AL, Ait-Ali T, Strait EL (2018). A novel inactivated vaccine against *Lawsonia intracellularis* induces rapid induction of humoral immunity, reduction of bacterial shedding and provides robust gut barrier function. Vaccine.

[17] Riber U, Heegaard PM, Cordes H, Stahl M, Jensen TK, Jungersen G (2015). Vaccination of pigs with attenuated *Lawsonia intracellularis* induced acute phase protein responses and primed cell-mediated immunity without reduction in bacterial shedding after challenge. Vaccine.

[18] Bak H, Rathkjen PH (2009). Reduced use of antimicrobials after vaccination of pigs against porcine proliferative enteropathy in a Danish SPF herd. Acta Vet Scand.

[19] Halloran K, Underwood MA (2019). Probiotic mechanisms of action. Early Hum Dev.

[20] Elshaghabee FMF, Rokana N, Gulhane RD, Sharma C, Panwar H (2017). Bacillus as potential probiotics: status, concerns, and future perspectives. Front Microbiol.

[21] Guedes RM, Gebhart CJ (2003). Comparison of intestinal mucosa homogenate and pure culture of the homologous *Lawsonia intracellularis* isolate in reproducing proliferative enteropathy in swine. Vet Microbiol.

[22] Lindecrona RH, Jensen TK, Andersen PH, Moller K (2002). Application of a 5′ nuclease assay for detection of *Lawsonia intracellularis* in fecal samples from pigs. J Clin Microbiol.

[23] Opriessnig T, Madson DM, Roof M, Layton SM, Ramamoorthy S, Meng XJ, Halbur PG (2011). Experimental reproduction of porcine circovirus type 2 (PCV2)-associated enteritis in pigs infected with PCV2 alone or concurrently with *Lawsonia intracellularis* or *Salmonella typhimurium*. J Comp Pathol.

[24] Bengtsson RJ, MacIntyre N, Guthrie J, Wilson AD, Finlayson H, Matika O, Pong-Wong R, Smith SH, Archibald AL, Ait-Ali T (2015). *Lawsonia intracellularis* infection of intestinal crypt cells is associated with specific depletion of secreted MUC2 in goblet cells. Vet Immunol Immunopathol.

[25] Mukaka MM (2012). Statistics corner: a guide to appropriate use of correlation coefficient in medical research. Malawi Med J.

[26] Goldberg RF, Austen WG, Zhang X, Munene G, Mostafa G, Biswas S, McCormack M, Eberlin KR, Nguyen JT, Tatlidede HS, Warren HS, Narisawa S, Millán JL, Hodin RA (2008). Intestinal alkaline phosphatase is a gut mucosal defense factor maintained by enteral nutrition. Proc Natl Acad Sci U S A.

[27] Penaloza-Vazquez A, Ma LM, Rayas-Duarte P (2019). Isolation and characterization of *Bacillus* spp. strains as potential probiotics for poultry. Can J Microbiol.

[28] Rhayat L, Maresca M, Nicoletti C, Perrier J, Brinch KS, Christian S, Devillard E, Eckhardt E (2019). Effect of *Bacillus subtilis* strains on intestinal barrier function and inflammatory response. Front Immunol.

[29] Fujiya M, Musch MW, Nakagawa Y, Hu S, Alverdy J, Kohgo Y, Schneewind O, Jabri B, Change EB (2007). The *Bacillus subtilis* quorum-sensing molecule CSF contributes to intestinal homeostasis via OCTN2, a host cell membrane transporter. Cell Host Microbe.

[30] Sumi CD, Yang BW, Yeo IC, Hahm YT (2015). Antimicrobial peptides of the genus *Bacillus*: a new era for antibiotics. Can J Microbiol.

[31] Hamley IW, Dehsorkhi A, Jauregi P, Seitsonen J, Ruokolainen J, Coutte F, Chataigné G, Jacques P (2013). Self-assembly of three bacterially-derived bioactive lipopeptides. Soft Matter.

[32] Gao XY, Liu Y, Miao LL, Li EW, Hou TT, Liu ZP (2017). Mechanism of anti-vibrio activity of marine probiotic strain *Bacillus pumilus* H2, and characterization of the active substance. AMB Express.

[33] Chowdhury SP, Hartmann A, Gao X, Borriss R (2015). Biocontrol mechanism by root-associated *Bacillus amyloliquefaciens* FZ. Front Microbiol.

[34] Le BC, Le NL, Formal M, Respondek F, Blat S, Apper E, Ferret-Bernard S, Le Huërou-Luron I (2017). Maternal short-chain fructo-oligosaccharide supplementation increases intestinal cytokine secretion, goblet cell number, butyrate concentration and *Lawsonia intracellularis* humoral vaccine response in weaned pigs. Br J Nutr.

[35] Leite FL, Vasquez E, Gebhart CJ, Isaacson RE (2019). The effects *of Lawsonia intracellularis*, *Salmonella enterica* serovar Typhimurium and co-infection on IL-8 and TNFα expression in IPEC-J2 cells. Vet Microbiol.

[36] Leite FLL, Singer RS, Ward T, Gebhart CJ, Isaacson RE (2018). Vaccination against *Lawsonia intracellularis* decreases shedding of *Salmonella enterica* serovar Typhimurium in co-infected pigs and alters the gut microbiome. Sci Rep.

